# Anaerobic Contribution Determined in Swimming Distances: Relation with Performance

**DOI:** 10.3389/fphys.2017.00755

**Published:** 2017-10-10

**Authors:** Eduardo Z. Campos, Carlos A. Kalva-Filho, Ronaldo B. Gobbi, Ricardo A. Barbieri, Nayara P. Almeida, Marcelo Papoti

**Affiliations:** ^1^Nucleus of Investigation in Sport Performance, Department of Physical Education, Federal University of Pernambuco, Recife, Brazil; ^2^Graduate Program in Motor Science, São Paulo State University, Rio Claro, Brazil; ^3^Graduate Program in Rehabilitation and Functional Performance, São Paulo University, Ribeirão Preto, Brazil; ^4^School of Physical Education and Sport, University of São Paulo, Ribeirão Preto, Brazil

**Keywords:** anaerobic capacity, swimming, performance, athletes, training

## Abstract

Total anaerobic contribution (TAn) can be assessed by accumulated oxygen deficit, and through sum of glycolytic and phosphagen contribution which enable the evaluation of TAn without influences on mechanical parameters. However, little is known about the difference of TAn within swimming distances. Therefore, the objectives of the present study were to determine and compare the TAn in different performances using the backward extrapolation technique and amount of lactate accumulated during exercise, and relate it with swimming performance. Fourteen competitive swimmers performed five maximal front crawl swims of 50, 100, 200, 400, and 800 m. The total phosphagen (AnAl) and glycolytic (AnLa) contributions were assumed as the fast component of post-exercise oxygen consumption (EPOC_FAST_) and amount of blood lactate accumulated during exercise, respectively. TAn was the sum of AnAl and AnLa. Significantly lower values of AnLa were observed in the 800 m (*p* < 0.01) than other distances. For AnAl, the 50 m performance presented the lowest values, followed by 100 and 800 m (*p* < 0.01). The highest values of AnAl were observed in the 200 and 400 m (*p* > 0.13). The TAn was significantly higher in the 200 and 400 m performances than observed at 50 and 800 m (*p* < 0.01). Anaerobic contributions were correlated with 50, 100, 200, and 400 m performances (*p* < 0.01). The AnAl contribution was not correlated with 400 m performance. Anaerobic parameters were not correlated with 800 m performance. In conclusion, the highest values of anaerobic contribution were observed in the 200 and 400 m distances. Moreover, TAn is important to performances below 400 m, and may be used in training routines.

## Introduction

Swimming performance depends on physiological (endurance capacity, and anaerobic fitness), technical, and morphological factors (Pelayo et al., 2007; Lätt et al., 2009; Kalva-Filho et al., 2015). In relation to physiological aspects, studies have demonstrated that both the aerobic and anaerobic metabolisms are important to swimming performance (Zamparo et al., 2000; Figueiredo et al., 2011; Kalva-Filho et al., 2015). Considering the large time volume expended during training to improve specific metabolisms, knowledge about energy balance in different performances is important for specific training prescription (Toussaint and Hollander, 1994).

Although the aerobic contribution seems to be easily calculated by the integral of oxygen consumption (VO_2_) during the effort (Figueiredo et al., 2011), the determination of the anaerobic contribution is complex. In this context, the most accepted method to estimate anaerobic contribution is the accumulated oxygen deficit (AOD), which is assumed as the difference between oxygen demand and aerobic contribution during an effort (Reis et al., 2010a,b; Kalva-Filho et al., 2016). However, at least three limitations may decrease its applicability for swimmer evaluations.

The first limitation is the time expended to determinate the oxygen demand, which requires several submaximal efforts performed on different days and at different intensities, decreasing the applicability of this method during training routines. The second limitation is that total phosphagen (AnAl) and glycolytic (AnLa) contributions cannot be determined separately using the AOD method, decreasing the possible investigation of these metabolisms in different swimming distances. Finally, the third limitation is the use of a snorkel and valve system to assess VO_2_ during swimming (Reis et al., 2010a,b), which reduces the speed and clearly disrupts the motor pattern, making undulations, turns, and side respiration impossible during the effort (Jalab et al., 2011; Campos et al., 2016). Considering the importance of these mechanical factors, the use of a snorkel and valve system could influence the AOD values and, consequently, lead to misinterpretation of the importance of the anaerobic metabolism in different performances.

In cycling, Bertuzzi et al. (2010) showed that AOD can be determined by the sum of AnAl, assumed as the fast component of post-exercise oxygen consumption (EPOC_FAST_), and AnLa, determined through the amount of blood lactate accumulated during exercise. These results have been confirmed by other authors (Zagatto et al., 2016a), allowing the use of this method in several modalities (Bertuzzi et al., 2015; Milioni et al., 2016; Zagatto et al., 2016b). Besides the determination of two anaerobic components, another advantage is the assessment of VO_2_ only after exercise, which eliminates the use of a snorkel and valve system.

In this context, the backward extrapolation technique consists of connecting the swimmer to a gas analyzer as quickly as possible after exercise (Montpetit et al., 1981; Costill et al., 1985). VO_2_ (log transformed) and recovery time are linearly adjusted and VO_2_ relative to the effort is determined at the end of exercise (Campos et al., 2016). Although the majority of studies have used the backward extrapolation technique in the first 30 s of recovery—assessing peak oxygen consumption (VO_2PEAK_) (Kalva-Filho et al., 2015; Campos et al., 2016)—the time of monitoring can be extended, allowing its use to determine EPOC_FAST_ (i.e., AnAl) (Kalva-Filho et al., 2015).

The advantages of determining anaerobic contributions through the sum of AnAl and AnLa, is maintaining the ecological validity of measurements, consequently increasing the applicability of results. However, as well as this parameter being frequently ignored in energy balance in swimming, few studies have used the backward extrapolation technique to determine AnAl values (Kalva-Filho et al., 2015). In addition, although these parameters have been extensively used to characterize different efforts, their comparison between different swimming distances, and the correlations of AnAl and AnLa with different swimming distances are scarce in swimming (Kalva-Filho et al., 2015). Thus, the objectives of the present study were to determine and compare the AnAl and AnLa in different swimming distances (50–800 m), using the backward extrapolation technique and the net of lactate appearance during exercise, and relate them with performance. Our hypothesis was that TAn would increase with swimming distance except for 800 m that would elicit minor TAn due to greater aerobic contribution.

## Materials and methods

### Participants

Fourteen competitive swimmers (seven male and seven female; age: 18.85 ± 3.18 years; body mass: 69.05 ± 12.3 kg) with 4 years minimum experience in national level competitions, volunteered in the present study. They were recruited at a swimming team of national level of a local university. This study was carried out in accordance with the recommendations of Declaration of Helsinki, approved by the Committee of Ethical on Research of São Paulo State University. All participants or they parents were informed about the experimental procedures, risk and benefits, and gave written informed consent. Participants were only recruited if they were not currently taking chronic or daily doses of anti-inflammatory medication and they were instructed to avoid drinking caffeine, alcohol, and energy drinks for at least 12 h prior to each measurement.

### Experimental design

Swimmers performed five maximal front crawl swims of 50, 100, 200, 400, and 800 m with an interval of at least 24 h between each bout. After a rest of 5 min, VO_2_ values at baseline were determined. Immediately after the efforts, a gas analyzer was connected to the swimmer (backward extrapolation technique) and VO_2_ was monitored during the first 5 min of recovery, to determine the EPOC_FAST_. Blood lactate concentrations ([La^−^]) were measured at baseline and during recovery, to establish the amount of accumulation.

Before each effort, a standardized warm up was performed consisting of ~1,000 m front crawl at low to moderate intensity, determined subjectively by the swimmers. The evaluations were performed in a 25-m swimming pool with water temperature of 26 ± 1°C. Expired gases were collected breath-by-breath (Quark PFT; Cosmed®, Rome, Italy). The gas analyzer was calibrated immediately before and verified after each test using a certified gravimetrically determined gas mixture. The ventilometer was calibrated pre-exercise and verified post-exercise using a 3 L syringe in accordance with the manufacturer's instructions. Following removal of outliers to exclude discrepant breaths, breath-by-breath data were interpolated (OriginPro 8.0, OriginLab Corporation, Microcal, Massachusetts, USA) to enhance the underlying responsive characteristics. Blood samples (25 μL) obtained from the ear lobe were used to determinate [La^−^] (YSI-2300; Yellow Springs Instruments®, Ohio, USA). Time of each effort was assessed manually through a digital chronometer (Casio, HS-70 W). Average speed was calculated by dividing distance by time of each bout.

### Backward extrapolation technique and blood lactate accumulation

After each effort, athletes were instructed to breathe immediately into a face mask (Hans Rudolph, Kansas City, MO, USA), connected to a breath-by-breath gas analyzer system (Quark PFT, Cosmed®, Rome, Italy). VO_2_ values were log-transformed and plotted against time, which was linearly adjusted. Thus, the y-intercept was considered as VO_2_ at the end of exercise (Montpetit et al., 1981; Bonne et al., 2014; Kalva-Filho et al., 2015; Campos et al., 2016), and assumed as the first point of recovery.

To calculate the amount of blood lactate accumulation, the [La^−^] was determined at baseline and the 3rd, 5, and 7th min during recovery. The [La^−^] at peak was measured during recovery and assumed as the highest value observed. The accumulation was the difference between [La^−^] at peak and baseline values (Δ[La^−^]).

### Anaerobic contributions

To calculate the EPOC_FAST_ values, VO_2_ during 5 min of recovery was adjusted by a bi-exponential decay fit (OriginPro 8.0, OriginLab Corporation, Microcal, Massachusetts, USA) (Bertuzzi et al., 2010; Zagatto et al., 2011; Kalva et al., 2015). Amplitude and time constant of the first exponential decay was determined and the product of these two variables was assumed as the EPOC_FAST_ or AnAl contribution (Bertuzzi et al., 2010; Zagatto et al., 2011; Kalva et al., 2015). AnLa was determined by the Δ[La^−^], considering a metabolic equivalent of 3 mL·O_2_·^−1^ kg^−1^ for each unit of lactate accumulated during the maximal effort (Zamparo et al., 2000). This metabolic equivalent has previously been used in swimming to determine AnLa (Zamparo et al., 2011; Kalva-Filho et al., 2015).

### Statistical analysis

The normality of data was previously confirmed using the Shapiro-Wilk's test, allowing presentation of data as mean ± standard deviation (*SD*). Mauchly's sphericity test was also applied, allowing the use of parametric statistics. The anaerobic parameters in different performances were compared through analysis of variance (ANOVA) for repeated measurements, followed by Tukey's *post-hoc* test. Pearson's correlation was used to test the relations between anaerobic parameters and performances in different distances. The correlation coefficient was classified as very small (0.00–0.19), small (0.20–0.39), moderate (0.40–0.69), strong (0.70–0.89), and very strong (0.90–1.00). For all analysis, the Statistical Package for Social Science software, version 17.0 (SPSS Inc, Chicago, Illinois) was used and the level of significance was *p* < 0.05.

## Results

Performance times in 50, 100, 200, 400, and 800 m were 28.7 ± 2.6, 64.3 ± 4.8, 137.2 ± 10.7, 292.1 ± 18.6, and 604.9 ± 45.6 s, respectively.

Parameters for anaerobic contribution determination in the different performances are demonstrated in Table 1. For AnAl parameters, the 50 m performance demonstrated the lowest values of amplitude (*p* < 0.001). On other hand, the highest values of time constant were observed after 50 and 200 m (*p* < 0.001). Although similar values for [La^−^] at baseline were observed (*p* > 0.08), peak values were lower after 50 and 800 m performances (*p* < 0.02). The lowest values of lactate accumulation were observed in the 800 m performance (*p* < 0.03) (Table 1).

**Table 1:** Parameters for anaerobic contribution determination at different performances.

	**Performances**				
	**50 m**	**100 m**	**200 m**	**400 m**	**800 m**
**AnAl**					
Amplitude (L.min^−1^)	2.0 ± 0.8	2.9 ± 0.9^a^	2.9 ± 0.7^a^	3.0 ± 1.1^a^	2.9 ± 0.9^a^
Time constant (min)	0.7 ± 0.1	0.6 ± 0.1^a^	0.8 ± 0.2^b^	0.6 ± 0.2^a^^c^	0.6 ± 0.1^a^^c^
**AnLa**					
[La^−^] at baseline (mM)	1.5 ± 0.6	1.7 ± 0.5	1.6 ± 0.3	1.9 ± 0.6	1.4 ± 0.3
[La^−^] at peak (mM)	9.9 ± 2.4^b^	10.8 ± 2.3^a^	12.2 ± 3.1^a^	12.0 ± 3.4^a^	7.8 ± 2.4^b^^d^^c^
[La^−^] accumulation (mM)	8.4 ± 2.4	9.1 ± 2.1	10.6 ± 3.3	10.0 ± 3.4	6.3 ± 2.5^a^^b^^d^^c^

*AnAl, anaerobic alactic contribution; AnLa, anaerobic lactic contribution; [La^−^], blood lactate concentration; [La^−^] accumulation, difference between blood lactate concentration at peak and baseline*.

a Significantly different from 50 m;

b Significantly different from 100 m;

c Significantly different from 200 m;

d *significantly different from 400 m*.

**Figure 1:**
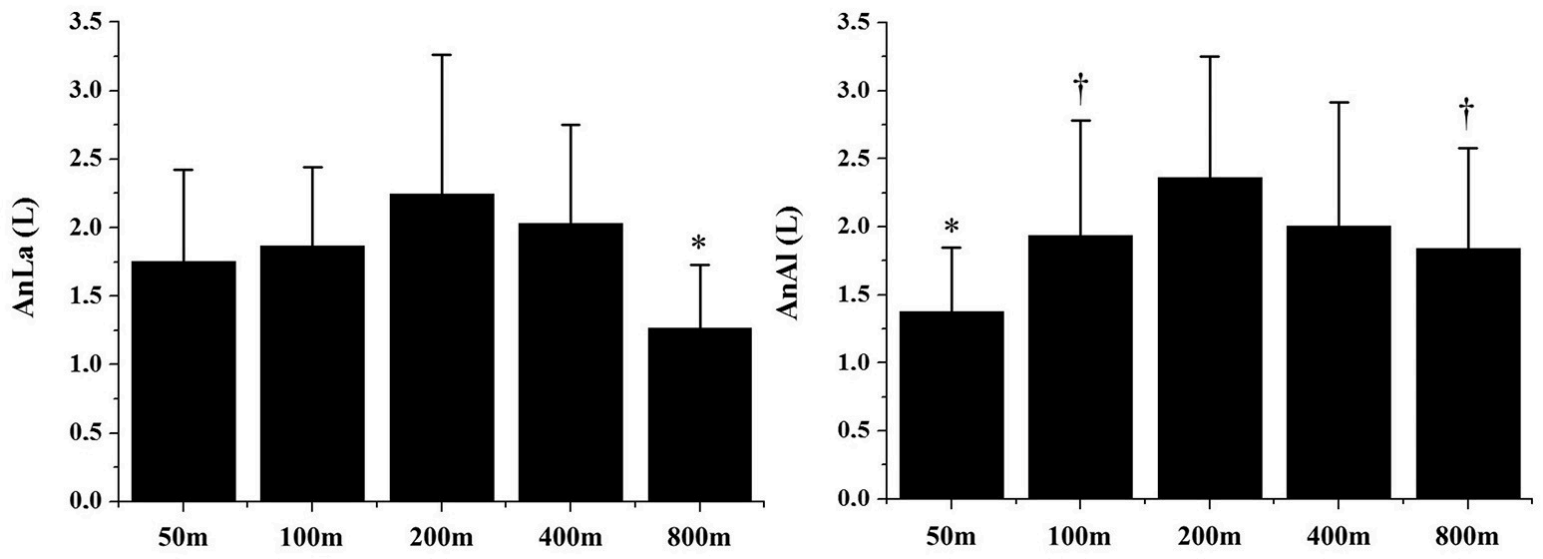
Alactic (AnAl) and lactic (AnLa) anaerobic contributions in 50, 100, 200, 400, and 800 m performances. ^*^Significant differences from other performances (*p* > 0.05); ^†^Significant differences from 200 to 400 m (*p* > 0.05).

**Figure 2:**
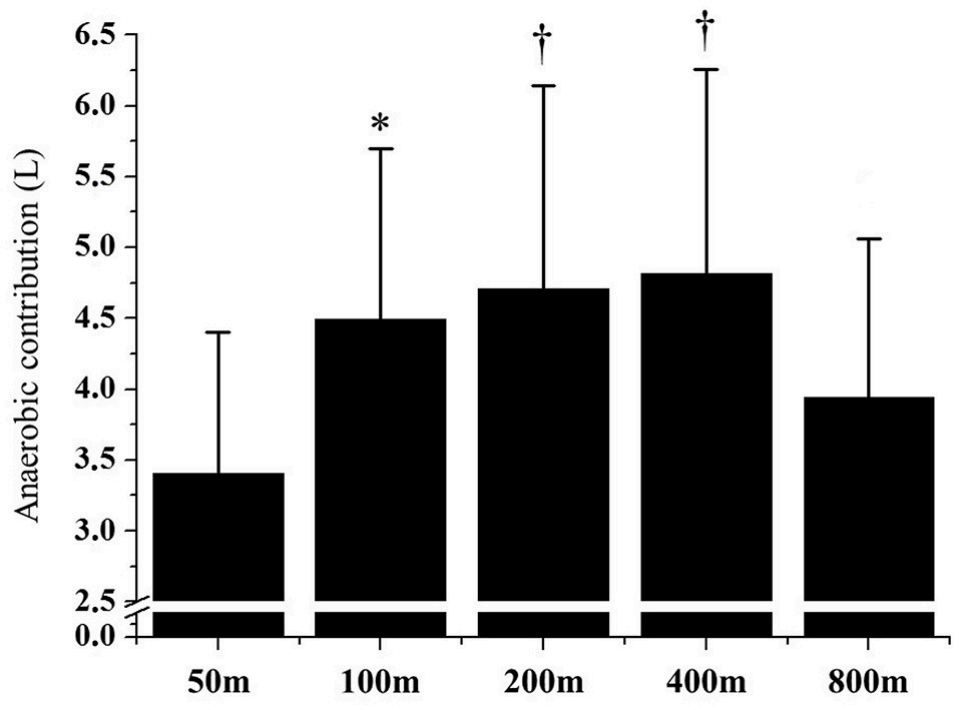
Anaerobic contribution in 50, 100, 200, 400, and 800 m performances. ^*^Significant differences from 50 m (*p* > 0.05); ^†^Significant differences from 50 to 800 m (*p* > 0.05).

Figure 1 demonstrates the AnAl and AnLa contributions in different performances. The AnLa contribution in the 800 m performance was significant lower than the 200 and 400 m performances (*p* < 0.01). No differences were observed between 50, 100, 200, and 400 m performances (*p* > 0.06). For AnAl contribution, values observed in the 50 m performance were significantly lower than in the 200 and 400 m performances (*p* < 0.01). No significant differences were observed between the longest performances (*p* > 0.13). Figure 2 demonstrates the total anaerobic contribution observed in different performances. No differences were observed between 100, 200, and 400 m performances (*p* > 0.70). The lowest values were observed during the 50 and 800 m performances, with no differences between these efforts (*p* = 0.20).

Table 2 demonstrates the correlations between physiological parameters and performances. Anaerobic contributions were correlated with 50, 100, and 200 m performances (*p* < 0.01). The AnAl contribution was not correlated with 400m performance. Anaerobic parameters were not correlated with 800 m performance.

**Table 2:** Correlation coefficients between physiological parameters and performances at different distances.

	**Performances**				
	**50 m**	**100 m**	**200 m**	**400 m**	**800 m**
AnAl	−0.64^*^	−0.74^*^	−0.87^*^	−0.45	−0.42
AnLa	−0.77^*^	−0.84^*^	−0.79^*^	−0.69^*^	−0.47
TAn	−0.91^*^	−0.85^*^	−0.88^*^	−0.68^*^	−0.53

AnAl, Alactic anaerobic contribution; AnLa, Lactic anaerobic contribution; TAn, Total anaerobic contribution;

* *Significant correlations (p < 0.05)*.

## Discussion

The present study aimed to compare anaerobic contribution determined in different distances, and relate it with swimming performance. The main findings of the present study were that AnLa contribution was lower in 800 m than 200 and 400 m, while no difference was found between 50, 100, 200, and 400 m. In relation to AnAl, 100, 200, 400, and 800 m did not present differences, and AnAl in the 50 m was significantly lower than in the200 and 400 m. The total anaerobic contribution was significantly higher in 100, 200, and 400 m, however, the 100 m TAn was similar to 800 m. Therefore, 200 and 400 m are the distances that present higher anaerobic contributions; both can be used to estimate the anaerobic capacity of swimmers, and also to assess changes arising from specific training. Finally, TAn can be assessed during swimming training periodization since it presents associations with performance (except 800 m).

The anaerobic contribution has been demonstrated to be important for swimming performance (Figueiredo et al., 2011; Kalva-Filho et al., 2015), thus its evaluation has clear meaning. However, the main limitation of anaerobic contribution determination is the need for time consuming tests - AOD (Reis et al., 2010a,b; Kalva-Filho et al., 2016)—performed with a snorkel, which reduces swimming speed (Jalab et al., 2011) and precludes undulations and turns. Therefore, the analysis of the anaerobic contribution through net lactate accumulation and VO_2_ recovery has several strengths over the classic method proposed for AOD determination, such as: (i) analysis of lactic and alactic contribution separately, (ii) enables undulations, turns, and side respiration, (iii) increases swimming speed, and (iv) is determined after one single effort.

The AnAl is determined through the product between the VO_2_ recovery amplitude and time constant (Margaria et al., 1933). We found that 50 m elicited significantly lower amplitude than other distances. This may be explained by the short effort time (28.7 ± 2.6 s), which was too short to increase VO_2_ to the same level as the other distances. The time constant was significantly higher in 200 m than other distances, except for 50 m. The amplitude of 200 m was similar to that observed by Sousa et al. (2013) in the same distance, however, the time constant was lower (27.03 ± 5.43 s vs. 48 ± 12 s, respectively). Thus, Sousa et al. (2013) observed an AnAl of 1.55 ± 0.13 L, which is significantly lower than observed in the present study. The likely reason for this difference is related to the use of a snorkel (Sousa et al., 2013), which may have reduced speed, increasing the aerobic contribution and decreasing the anaerobic contribution. This hypothesis is sustained by the similar results observed in AnAl contribution over 400 m in the study of Kalva-Filho et al. (2015) (Amplitude: 3.0 ± 1.3 L and Time constant: 0.9 ± 0.3 s), since they also used free swimming performance, which does not limit the athletes mechanics and speed, increasing the anaerobic contribution.

The 200 and 400 m efforts elicited higher AnAl than the other distances. One possible reason for this result is that athletes perform 50 to 400 m performances above their critical speed, which does not enable recovery of creatine phosphate (Jones et al., 2008) and thus during 200 and 400 m athletes execute more turns at high intensity. It is important to note that, besides no statistical difference, the mean 400 m AnAl was lower than 200 m, which may indicate that, although athletes execute more turns at 400 m than at 200 m, the turns are performed at lower intensity. The lower value observed at 50 and 100 m may also indicate that the time spent in high intensity effort may contribute to AnAl. Future studies may want to correlate AnAl and mechanical parameters related to turns and strokes to confirm this hypothesis.

In relation to AnLa, the baseline [La^−^] was similar between efforts. This result is important as AnLa takes into account the difference between peak and baseline [La^−^] (di Prampero and Ferretti, 1999). In the present study, the 800 m presented lower values of AnLa than the other distances. These results can also be explained by the fact that the 800 m effort is performed at intensities close to critical speed, which reduces lactic anaerobic contribution. The accumulation found in the 800 m effort may be explained since athletes usually increase their speed at the end of the effort. Mauger et al. (2012) observed that swimmers use different pacing strategies during a 400 m effort, however, no studies have investigated whether these strategies may interfere in the aerobic or anaerobic contribution.

Total anaerobic contribution was higher at 100, 200, and 400 m than at the other distances, without differences between then. The TAn found in the present study for 100 m (55.12 ± 12.65 mL·kg^−1^), 200 m (65.74 ± 16.18 mL·kg^−1^), and 400 m (58.80 ± 16.50 mL·kg^−1^) were significantly higher than in the study of Reis et al. (2010a) (20.55± 7.41 mL·kg^−1^, 17.53 ± 13.53 mL·kg^−1^, and 11.91 ± 14.72 mL·kg^−1^, respectively. The main reason for this difference was the method used to estimate TAn. The Reis study used the AOD method to estimate anaerobic contribution, which requires a snorkel to analyze the differences between theoretical demand and aerobic demand. The mean speed during the 100, 200, and 400 m efforts were lower in the study of Reis et al. (2010a) compared with the present study, even though their swimmers were only male and of a higher level. This may indicate that, when effort is performed with a snorkel, swimmers may be obligated to reduce speed, increasing the aerobic contribution and reducing the anaerobic contribution. These results highlight that the use of the backward extrapolation technique and net lactate appearance may be more feasible and present greater ecological validity than the accumulated oxygen deficit method to evaluate TAn in swimming. Furthermore, although other studies may want to compare TAn evaluated by accumulated oxygen deficit and the method used in the present study, due to this mechanical difference, comparison between them would be limited.

The possibility of determining AnAl and AnLa to estimate TAn is also important to verify their possible relation with swimming performance. Kalva-Filho et al. (2015) recently showed that TAn is highly associated with 400 m performance, however a relation of AnAl and AnLa with performance was not observed. We did not find any association of anaerobic contribution with 800 m performance which demonstrates that this distance is highly dependent on the aerobic metabolism. We found a significant association between AnAl and 50, 100, and 200 m performances, without a relation with 400 m. This clearly explains that this metabolism is determinant for short distance swimming performance. AnLa associates with 50 to 400 m performance, as well as TAn. It is interesting to note that, even though 50 m elicited lower values of TAn than 100, 200, and 400 m, the relation of this variable with 50 m performance was stronger than the others (*r* = −0.91; Table 2), which indicates that the 50 m effort is highly dependent on the anaerobic contribution. Therefore, the use of the backward extrapolation technique together with the net lactate accumulation is a feasible method to calculate anaerobic contribution in swimming, and may be used during swimming training periodization in order to determine whether training stimulation are effective in elicit changes in these variables. Athletes and coaches may use these results during training prescription for different athletes, according to TAn, and, specially, AnAl and AnLa. Finally, if coaches wish to evaluate swimmers' maximal anaerobic contribution, they should use distances between 200 and 400 m.

The strengths of the present study are: (i) swimmers were able to swim freely and perform they maximal effort without snorkel interference, and (ii) we evaluate TAn separately which gives an overview of energy system contribution during swimming. The main limitation of the present study is the use of both genders, since women seem to have lower anaerobic capacity than man (Hunter, 2014) thus, further studies should investigate the differences between men and women with a larger sample size. The evaluation of energy contribution during swimming distances provides a view of athlete's metabolic behavior during simulated trial, and since we did not control the strategy used during the effort—which might modify swimming TAn—the generalization to other athletes must be done with caution. Therefore, future researches may want to investigate whether TAn (i.e., AnAl and AnLa) changes with modification in swimming strategy among different swimming distances.

Considering this limitation, AnAl is higher in 200 and 400 m than the other distances, mainly due to the time spent at high speed, the AnLa is similar between the distances, however 800 m presented low values of lactate accumulation. The TAn is higher in 100, 200, and 400 m than the other distances, and coaches may want to select 200 and/or 400 m to evaluate the maximal anaerobic contribution of swimmers. Besides its feasibility, AnAl, AnLa, and TAn are highly associated with performance, and can be used in training routines, in order to assess modifications in TAn over the season.

## Author contributions

EC: Data acquisition; data interpretation; manuscript writing. CK: Data interpretation; manuscript writing; Manuscript revision. RG: Data acquisition; manuscript writing. RB: Data acquisition; data interpretation; manuscript revision. NA: Data acquisition; data interpretation. MP: Data analysis; manuscript revision; conception and study design; final approval of the version.

### Conflict of interest statement

The authors declare that the research was conducted in the absence of any commercial or financial relationships that could be construed as a potential conflict of interest.
